# La plastie tricuspide: annuloplastie de Carpentier versus technique de De VEGA

**DOI:** 10.11604/pamj.2017.27.119.8868

**Published:** 2017-06-15

**Authors:** Salma Charfeddine, Rania Hammami, Faten Triki, Leila Abid, Mourad Hentati, Imed Frikha, Samir Kammoun

**Affiliations:** 1 Service de Cardiologie, Hôpital Hedi Chaker, SFAX, Tunisie; 2 Service de Chirurgie Cardio-Vasculaire, Hôpital Habib Bourguiba SFAX, Tunisie

**Keywords:** Fuite, valve tricuspide, annuloplastie, Leakage, tricuspid valve, annuloplasty

## Abstract

L'atteinte de la valve tricuspide a été longtemps négligée aussi bien par cardiologue que par le chirurgien, mais depuis quelques années, la fuite tricuspidienne a été démontrée comme un facteur pronostic dans l'évolution des patients opérés d'une valvulopathie du c'ur gauche. Plusieurs techniques de plastie tricuspide ont été développées et les études publiées divergent sur les résultats de ces techniques; Nous avons mené ce travail afin d'évaluer les résultats de plastie tricuspide dans une population caractérisée par une forte prévalence de la maladie rhumatismale et de comparer les techniques d'annuloplastie de carpentier versus la plastie de DeVEGA. Etude rétrospective menée sur une période de 25 ans ayant inclus les patients traités par plastie tricuspide dans le service de cardiologie de SFAX. Nous avons comparé les résultats de groupe 1 (annuloplastie de Carpentier) vs groupe 2 (plastie de DeVEGA) 91 patients étaient inclus dans notre étude, avec 45 patients dans le groupe 1et 46 patients dans le groupe 2. La plupart des patients avaient une IT moyenne ou importante (83%) avant la chirurgie, une dilatation de l'anneau a été observée chez 90% des patients sans qu'il y ait une différence significatives entre le groupe 1 et 2. Les résultats immédiats étaient comparables entre les deux techniques mais au cours de suivi une insuffisance récurrente au moins moyenne a été plus significativement fréquente dans le groupe de plastie de DeVEGA. Les facteurs prédictifs d'IT récurrente significative au long cours étaient en étude multivariée la technique de DeVEGA (OR=3.26 (1.12-9.28)) et la pression artérielle pulmonaire systolique préoperatoire (OR=1.06 (1.01-1.12)). La plastie tricuspide avec anneau de Carpentier semble garantir de meilleurs résultats que la plastie de DeVEGA, par contre une PAPS préopératoire élevée est prédictive de récurrence de la fuite tricuspidienne même après plastie d'où l'intérêt d'opérer les malades à un stade précoce.

## Abstract (en)

Tricuspid valve disease has been neglected for a long time by cardiologists and surgeons, but for some years now leakage of tricuspid valve has been demonstrated as a prognostic factor in the evolution of patients with left heart valve disease undergoing surgery. Several techniques for plastic repair of tricuspid valve have been developed and the published studies differ on the results of these techniques; we conducted this study to assess the results of plastic repair of tricuspid valve in a population of patients with a high prevalence of rheumatic disease and to compare Carpentier's ring annuloplasty techniques with DEVEGA plasty. We conducted a retrospective study of patients undergoing plastic repair of tricuspid valve in the Department of Cardiology at the Medicine University of Sfax over a period of 25 years. We compared the results from the Group 1 (Carpentier's ring annuloplasty) with Group 2 (DeVEGA plasty). 91 patients were included in our study, 45 patients in the Group 1 and 46 patients in the Group 2. Most patients had mean or severe TI (83%) before surgery, ring dilation was observed in 90% of patients with no significant difference between the two groups. Immediate results were comparable between the two techniques but during monitoring recurrent, at least mean, insufficiency was significantly more frequent in the DeVEGA plasty Group. The predictive factors for significant recurring long term TI were DeVEGA technique (OR=3.26[1.12-9.28]) in multivariate study and preoperative pulmonary artery systolic pressure (OR=1.06 (1.01-1.12)). Plastic repair of tricuspid valve using Carpentier's ring seems to guarantee better results than DeVEGA plasty. On the other hand, preoperative high PASP is predictive of recurrent leakage of tricuspid valve even after plasty; hence the importance of surgery in the treatment of patients at an early stage of the disease.

## Introduction

L'atteinte valvulaire tricuspide est la plus rare de toutes les valvulopathies; elle est le plus souvent associée ou secondaire à une atteinte valvulaire gauche mitrale ou mitro-aortique. Dans le cadre du rhumatisme articulaire aigu (RAA), l'atteinte de la valve tricuspide peut être organique et/ou fonctionnelle. En effet, la valve tricuspide devient souvent incontinente par dilatation des cavités cardiaques droites et de l'anneau tricuspide sans qu'il y ait des lésions anatomiques des valves. Considérée pendant longtemps comme «une valve orpheline», des études pionnières se sont intéressées à cette valvulopathie et ont montré une régression de l'insuffisance tricuspide (IT) fonctionnelle après correction des valvulopathies gauches [1]. Des études plus récentes sont venues contre-indiquer ces données, en soulignant l'impact pronostique à court et à long terme de la correction tricuspide associée au cours du geste opératoire. En effet, une IT peut s'aggraver ou même se développer de novo après une chirurgie du c'ur gauche isolée [2, 3]. L'IT non corrigée a été démontrée comme un facteur de mauvais pronostic avec une morbi-mortalité élevéechez les patients opérés pour une valvulopathie du c'ur gauche [4, 5]. De ce fait, le t'appareil altéré. La technique de la réparation est encore un sujet de débat. Selon certains chirurgiens, la plastie tricuspide avec un anneau est associée à de meilleurs résultats [6–8], tandis que d'autres ont démontré des résultats plutôt en faveur de la plastie basée sur la simple suture [9, 10]. Les objectifs de cette étude étaient de comparer les différentes techniques de plastie tricuspide, préciser les modalités post opératoires immédiates et tardives, dégager les facteurs pronostiques prédictifs de l'IT récurrente significative au cours du suivi post opératoire.

## Méthodes

Il s'agit d'une étude rétrospective, réalisée durant la période allant de 1990 jusqu' à la fin de l'année 2014, et incluant tous les patients ayant bénéficié d'une plastie tricuspide et suivis dans le centre de cardiologie de l'hôpital Hédi Chaker de Sfax. La valvulopathie tricuspide peut être d'origine organique fuyante et/ou sténosante correspondant à une atteinte des valves par le processus rhumatismal et/ou d'origine fonctionnelle, significative, par dilatation de l'anneau tricuspide, secondaire au retentissement des valvulopathies gauches sur les cavités droites. Tous les patients inclus dans cette étude ont bénéficié d'une échographie cardiaque trans-thoracique (ETT) pré et post opératoire afin de quantifier les différentes valvulopathies, d'évaluer la fonction ventriculaire gauche et droite. Une dysfonction ventriculaire droite (VD) échocardiographique a été définie par une valeur de l'excursion à l'anneau tricuspide (TAPSE) < 15 mm et une onde S à l'anneau tricuspide latéral par le doppler tissulaire < 10 cm/sec. La réparation tricuspide a consisté soit en une annuloplastie par un anneau de Carpentier-Edwards (Groupe 1) soit une plastie de De-Vega (Groupe 2). Une IT post opératoire significative a été définie par une fuite au moins moyenne.

**Analyse statistique:** les variables ont été exprimées sous forme de moyenne ± écart type ou de pourcentage selon qu'ils s'agissaient de données quantitatives ou qualitatives. Les comparaisons de 2 moyennes sur séries indépendantes ont été effectuées au moyen du test t de Student. Les comparaisons de plusieurs (> 2) moyennes sur séries indépendantes ont été effectuées au moyen du test F de Snedecor d'analyse de la variance paramétrique (ANOVA à un facteur). Les comparaisons de pourcentages sur séries indépendantes ont été effectuées par le test du chi-deux de Pearson. Les relations entre les variables quantitatives ont été recherchées par la méthode de régression linéaire. Une différence est considérée comme significative lorsque p < 0.05. Le test de Pearson était employé pour rechercher une corrélation significative. Une corrélation positive était considérée comme forte si supérieure à 0.5 avec une valeur de p inférieure à 0.05 pour affirmer que cette corrélation n'est pas due au hasard. Le développement d'une IT significative durant le suivi a été évalué selon le modèle de régression de COX. Les analyses statistiques ont été réalisées avec le logiciel PASW^®^ statistics version 20 (SPSS inc, Chicago, Illinois, USA).

## Résultats

Au cours de la période de l'étude, 91 patients ont bénéficié d'une plastie tricuspide dans notre centre. L'âge moyen de ces patients était de 43 ± 12 ans avec des extrêmes allant de 16 à 71 ans. Nos patients se répartissaient en 66 (72.8%) femmes et 25 (27.2%) hommes. Six patients (6.5%) avaient bénéficié d'au moins une dilatation mitrale percutanée (DMPC) et 17 patients (18.6%) avaient des antécédents de chirurgie valvulaire cardiaque (plastie tricuspide réalisée dans le cadre de chirurgie rédux). Aucun de nos patients n'a eu auparavant un geste chirurgical sur la valve tricuspide. L'expression clinique était polymorphe chez nos patients dont la plupart pluri-valvulaire. Ainsi au cours de leur hospitalisation, 48 patients (52.7%) avaient une dyspnée d'effort selon la classification de la New York Heart Association (NYHA) III, 17 patients (18.7%) avaient une dyspnée de classe NYHA IV et 45 patients (49.4%) avaient des palpitations. Soixante-seize patients (83.5%) étaient en arythmie complète par fibrillation auriculaire (ACFA) permanente. La fuite tricuspide était modérée chez 15 patients (16.5%), moyenne chez 27 patients (29.7%) et sévère chez 49 patients (53.8%). Une dilatation de l'anneau tricuspide a été noté chez 82 patients (90%). La plastie tricuspide consistait en une annuloplastie par un anneau de Carpentier-Edwards chez 45 patients (49.5%) et une annuloplastie selon la méthode de De-Vega chez 46 patients (50.5%). La plastie tricuspide était associée à un remplacement valvulaire mitral isolé chez 59 patients (64.8%), à un remplacement valvulaire aortique isolé chez 3 patients (3.3%) et à un double remplacement valvulaire mitro-aortique chez 26 patients (28.6%). Aucun geste isolé sur la tricupside n'é été réalisé dans notre série. Nous n'avons pas noté de différence significative entre les 2 groupes de patients ayant une annuloplastie par un anneau de Carpentier ou une plastie de De-Vega en ce qui concerne l'âge, le sexe, la classe fonctionnelle selon la NYHA, le grade de l'IT en préopératoire, la fraction d'éjection du ventricule gauche (FEVG) ou la pression artérielle pulmonaire systolique (PAPS) (Tableau 1).

**Tableau 1:** Caractéristiques préopératoires dans la population d’étude selon le type de la plastie tricuspide

Caractéristiques	Population totale		Groupe 1 (annuloplastie de carpentier)		Groupe 2 (plastie de DEVEGA)		p
	nombre	%	nombre	%	nombre	%
**Nombre de patients**	91		45	49.5	46	50.5	NS
Age (ans ± ET)	43.58 ± 12.56		44.11 ± 11.63		43.17 ± 13.34		NS
Sexe féminin	66	72.8	28	62.22	31	67.39	NS
**NYHA préopératoire**							
NYHA III	48	52.7	24	53.33	24	52.17	NS
NYHA IV	17	18.7	6	13.33	11	23.91	NS
FA chronique	76	83.5	38	84.44	38	82.60	NS
**Grade de l’IT préopératoire**							
Modérée	15	16.5	9	20	6	13.04	NS
Moyenne	27	29.7	13	28.88	14	30.43	NS
Sévère	49	53.8	23	51.11	26	56.52	NS
Dysfonction VD préopératoire	18	19.78	5	11.11	13	28.26	0.04
Taille de l'anneau tricupide (mm)	44±7		44,9±9		43,1±8		NS
dilatation de l'anneau>40 mm	82	90	40	88,8	42	91,3	NS
FEVG préopératoire (%)	57.29 ± 5.82		57.26 ± 5.75		57.32 ± 5.95		NS
PAPS préopératoire (mmHg)	49.63 ± 10.82		49 ± 10.79		50.26 ± 10.93		NS
Chirurgie redux	17	18.6	7	15.55	10	21.73	NS
**PT isolée**	0						
RVM + PT	59	64.8	29	64.44	30	65.21	NS
RVA + PT	3	3.3	1	2.22	2	4.34	NS
RVM + RVA + PT	26	28.6	12	26.66	14	30.43	NS
PAC + RV + PT	3	3.3	2	4.44	1	2.17	NS

NYHA: New York heart association, FA: fibrillation auriculaire, IT: insuffisance tricuspide, VD: ventricule droit, FEVG: fraction d’éjection du ventricule gauche, PAPS: pression artérielle pulmonaire systolique, RVM: remplacement valvulaire mitral, RVA: remplacement valvulaire aortique, PT: plastie tricuspide, PAC: pontage aorto-coronaire

En post opératoire immédiat, nous avons noté une amélioration significative du grade de la fuite tricuspide (Tableau 2). Seulement 12 patients (13.2%) avaient une IT résiduelle significative (moyenne ou sévère) sans qu'il y ait de différence statistiquement significative entre les 2 groupes de patients (groupe 1=13.3% vs groupe 2= 13.1%, p=NS). Vingt-deux patients (24.2%) avaient une IT résiduelle modérée en post opératoire. La PAPS préopératoire (54.63 ± 10.40 vs 45.54 ± 9.41, p<0.001) et la PAPS postopératoire (36.89 ± 12.67 vs 32.23 ± 8.78, p=0.04) étaient significativement plus élevées chez les patients avec une IT résiduelle significative. Le délai moyen de suivi post opératoire de ces patients était de 38 ± 16,8 mois soit 3.16 ans. Cinq patients (5.4%) sont décédés au cours du suivi: 3 patients (3.3%) sont décédés en post opératoire immédiat, deux d'entre eux ont développé un bloc auriculo-ventriculaire complet avec un état de choc cardiogénique, le 3ème patient est décédé suite à un état de choc hémorragique. Deux autres patients sont décédés au cours du suivi tardif suite une endocardite infectieuse mitrale compliqué d'état de choc septique et cardiogénique. Trente patients (33 %) ont été ré-hospitalisés durant le suivi. Parmi ces patients, 8 (8.8%) ont présenté un ou plusieurs épisodes d'insuffisance cardiaque et 3(3.3 %) ont présenté un ou plusieurs épisodes d'insuffisance cardiaque droite au cours de leur suivi. Huit patients (8.8%) ont présenté une endocardite infectieuse sur prothèse mitrale. Au cours du suivi post opératoire tardif, 31 patients (21.9%) ont développé ou aggravé leurs fuites devenant une fuite significative (moyenne ou sévère) chez 20 patients (44.4%) dans le groupe annuloplastie de De-Vega et 11 patients (23.9%) dans le groupe annuloplastie avec un anneau de Carpentier avec une différence statistiquement significative (p = 0.05). En étude univariée, les patients ayant développé une IT significative (31 patient, soit 34%) au cours du suivi avaient significativement un anneau tricuspide plus dilaté, une PAPS préopératoire plus élevée et ils ont plutôt bénéficié plus fréquemment d'une plastie de DeVega (Tableau 3). En étude multivariée, le degré de la PAPS préopératoire ainsi que la technique de plastie tricuspide étaient identifiées comme des facteurs prédictifs indépendants de la récidive d'une fuite tricuspide au moins moyenne (Tableau 4).

**Tableau 2:** Comparaison de l’importance de la fuite tricuspide dans la population d'étude avant et après la chirurgie

Grade de l’IT	Avant la chirurgie	Après la chirurgie	Valeur p
IT absente ou minime	0	57 (62.6%)	< 0.001
IT modérée	15 (16.5%)	22 (24.2%)	NS
IT moyenne	27 (29.5%)	4 (4.4%)	< 0.001
IT importante	24 (53.8%)	8 (8.8%)	< 0.001

IT: insuffisance tricuspide

**Tableau 3:** Facteurs prédictifs de développement d’une IT significative au cours du suivi dans la population d’étude en étude uni-variée

	IT significative secondaire (31 patients)	Pas d’IT significative secondaire (60 patients)	Valeur p
Age	44,5±14	43±11	0.62
Sexe féminin	23 (74,2%)	46 (76,7%)	0,49
Fa chronique	24 (77,4%)	52 (86,7%)	0,2
IT préopératoire importante	18 (58,1%)	31 (51,7%)	0,36
Dysfonction VD préopératoire	8 (25,8%)	10 (16,7%)	0,22
FeVG postopératoire (%)	54,2±6	61±6	0,52
Diamètre de l’anneau en préopératoire (mm)	43,4±3	41,2±2,8	0,002
PAPS préopératoire (mmHg)	54,8±9	46,9±10	0,001
PAPS postopératoire (mmHg)	35,9±10	33±10	0,32
Technique de Plastie			
*Plastie de DeVEGA	20 (64,5%)	26(43,3%)	0,05
*Annuloplastie de carpentier	11 (35,4%)	34 (55,6%)	

Fa: fibrillation atriale, IT: insuffisance tricuspide, VD: ventricule droit, FeVG: fraction d’éjection du ventricule gauche, PAPS: pression artérielle pulmonaire systolique

**Tableau 4:** Facteurs prédictifs de développement d’une IT significative au cours du suivi dans la population d’étude en étude multi-variée

Facteur de risque	Odds ratio [IC 95%]	Valeur p
FA chronique	1.42 [0.4-5.06]	0.58
l’IT préopératoire importante	0.52 [0.12-2,08]	0.12
FEVG postopératoire	0.94 [0.97-1.01]	0.63
**PAPS préopératoire**	1.06 [1.01-1.12]	**0.015**
Dysfonction VD préopératoire	0.92 [0.27 – 3.06]	0.27
PAPS postopératoire	1.02 [0.98-1.07]	0.23
**Plastie de DEVEGA**	3.26 [1.12-9.48]	**0.03**

Fa: fibrillation atriale, IT: insuffisance tricuspide, VD : ventricule droit, FeVG: fraction d’éjection du ventricule gauche, PAPS: pression artérielle pulmonaire systolique

## Discussion

L'atteinte tricuspide est la plus rare de toutes les valvulopathies, elle est le plus souvent associée ou secondaire à une atteinte valvulaire du cœur gauche [11]. L'âge moyen de nos patients était de 43.58 ± 12.56 ans. L'âge moyen de nos patients était similaire à celui rapporté dans les trois séries Tunisiennes précédentes [11–12]. Notre population d'étude était moins âgée comparativement aux études Européennes et Américaines [7, 8, 10, 13]. Une prédominance féminine a été retrouvée dans notre série (72.8%) et rapportée par la majorité des études publiées précédemment variant de 62 à 83% [6, 11, 14]. Nos patients présentaient une atteinte polyvalvulaire. L'origine rhumatismale explique cette atteinte multi valvulaire ainsi que le jeune âge. Soixante de nos patients (65.9 %) avaient une triple atteinte valvulaire (mitrale, aortique et tricuspide) dont 26 (28.6%) ont nécessité un triple remplacement valvulaire. Ce taux était de 56% dans l'étude de Ben Ameur et al. [11] et de 60% dans l'étude de Bernal et al. [14]. Dans notre étude, 18.6 % des patients antécédents de chirurgie cardiaque. Ce taux était moindre que celui rapporté dans l'étude de Ben Ameur (37%) et à celui de Bernal (26%) [11, 14]. Pendant longtemps, la valve tricuspide était ignorée au cours de la chirurgie valvulaire gauche. Initialement, de bons résultats post opératoires ont été observés après chirurgie mitrale et/ou aortique isolée sans geste sur la tricuspide, même en présence d'une IT importante [1, 15]. Cette attitude a été ensuite reconsidérée. En effet, de nombreuses publications récentes ont démontré que l'IT pourrait s'aggraver ou apparaitre de nouveau malgré la correction des valvulopathies gauches [3, 16]. De même, la persistance d'une IT significative était associée à un mauvais pronostic à court et à long terme [5, 6, 17]. Actuellement, un geste sur la valve tricuspide associé à la chirurgie valvulaire gauche est fortement recommandé selon les publications des sociétés savantes [18–20]. La stratégie thérapeutique et le choix de la méthode chirurgicale sont conditionnés par l'état clinique du patient, les données précises de l'échocardiographie préopératoire et de l'échocardiographie trans-oesophagienne per opératoire [21] ainsi que les remaniements anatomiques de la valve tricuspide. Beaucoup de techniques de réparation avec ou sans annuloplastie tricuspide ont été décrites [8]. Chez la majorité des patients, l'IT est fonctionnelle avec un défaut de coaptation des feuillets valvulaires sans remaniements valvulaire. Par conséquent, la réparation chirurgicale s'est intéressée principalement à l'anneau tricuspide, bien qu'il y ait un débat en cours sur la technique de la réparation avec ou sans anneau prothétique [13]. Dans notre étude, nous avons démontré une amélioration significative du degré de la fuite tricuspide après la plastie en postopératoire immédiat. Seulement 12 patients (13.2%) avaient une IT résiduelle significative (moyenne ou sévère) quel que soit le type de la plastie effectuée avec ou sans anneau prothétique. Ce taux était similaire à celui rapporté dans des séries précédentes. Ghanta et al. [10] ont rapporté environ 8% d'échec de la chirurgie avec une IT significative résiduelle dans une population ayant bénéficié d'une plastie avec ou sans un anneau prothétique. De même, dans l'étude de McCarthy et al. [6] à propos de 790 patients, le taux d'IT résiduelle significative était de 14% quel que soit le type de l'annuloplastie.

Parmi les annuloplasties à base de suture, la technique de De Vega [22] est le plus couramment utilisée car elle préserve l'anatomie et la flexibilité de l'espace annulaire. En plus, elle a comme avantages l'absence de matériel étranger, donc un coût très faible et un temps opératoire bref [23, 24]. La suture avec bicuspidisation initialement décrite par Kay [25] est actuellement moins effectuée. Certaines séries ont démontré de bons résultats à court et à long terme avec l'annuloplastie tricuspide avec les techniques de DeVega et de Kay [9, 10]. Cependant, d'autres auteurs ont mis en doute ces résultats devant l'incidence élevée d'IT récidivante au cours du suivi, en particulier en présence d'une hypertension artérielle pulmonaire et d'une dilatation importante de l'anneau tricuspide [6, 26]. L'annuloplastie tricuspide avec un anneau prothétique a été initialement proposée devant une IT récidivante. L'anneau flexible de Carpentier a été démontré supérieur à l'annuloplastie de De Vega dans une des rares études randomisées réalisées sur ce type de chirurgie [27]. Ceci est en particulier vrai en absence de lésions organiques de la valve tricuspide [26]. Ces résultats ont été confirmés par d' autres études [7, 13]. Une méta-analyse récente [28] a effectivement bien démontré la supériorité de l'annuloplastie par un anneau de Carpentier à long terme sur l'apparition d'IT postopératoire. En effet, les résultats selon les courbes de survie étaient superposables au début, mais devenaient nette récidivante significative à environ 3 ans de suivi, avec une différence statistiquement significative en faveur de l'annuloplastie avec un anneau prothétique. Les facteurs de risque associés au développement d'une IT significative étaient essentiellement l'hypertension artérielle pulmonaire en pré quel que soit le type de l'annuloplastie. Ceci a été déjà rapporté dans plusieurs études [10, 17]. En effet, ceci pourrait être expliqué par un degré plus important de dysfonctionnement de la valve tricuspide avec une dilatation de l'anneau tricuspide ou un tenting valvulaire plus important, et ainsi plus de difficulté de réparation valvulaire [29]. De plus, il ressort dans notre série que l'annuloplastie semi-circulaire à la De Vega constitue un autre facteur d'échec de la plastie tricuspide; mais ceci n'est statistiquement significatif, dans notre étude, qu'au-delà de 3 ans de suivi, ce qui rejoint en partie les résultats retrouvés par McCarthy [6]. Cette récidive est parti¬culièrement secondaire au phénomène de «Gliding» ou «Guitar string» (effet rideau) responsable de déhiscence des sutures à long terme [30]. De ce fait, ces auteurs recommandent l'abandon des annuloplasties non prothétiques les considérant comme des facteurs de risque d'échec de la réparation tricuspide. D'autres auteurs continuent à réparer les fuites tricuspides fonctionnelles par le procédé de De Vega, attestant de leurs très bons résultats à court, moyen et long termes [22, 24].

## Conclusion

Le bénéfice d'une correction initiale systématique des fuites tricuspides précocement dans le même temps opératoire que le geste valvulaire gauche a été largement démontré. L'apparition tardive d'une insuffisance tricuspide fonctionnelle au cours du suivi représente un facteur de mauvais pronostic dans l'évolution de ces patients. Ainsi, la technique de la plastie tricuspide a été pendant longtemps un sujet de débat. En cas d'annuloplastie, les anneaux rigides ou semi-rigides donnent, au long cours, de bons résultats, nettement meilleurs que ceux de l'annuloplastie à la De Vega.

### Etat des connaissances actuelle sur le sujet

- L'importance de traiter la valve tricuspide chez les patients opérés du cœur gauche puisque cette atteinte influence de proche le pronostic à long terme et surtout la qualité de vie du patient;
- Dans la littérature, il y a des publications disparates concernant les résultats des différentes techniques de plastie tricuspide: bien évidemment une grande partie de ces études étaient en faveur de la plastie avec un anneau de carpentier.

### Contribution de notre étude à la connaissance

- La quasi majorité des études publiées, concernaient des pays développés caractérisés par la prédominance de l'atteinte dégénérative: cette étude découle d'une population caractérisée par la prédominance de l'atteinte rhumatismale, qui certes influence différemment l'évolution de la fuite et la plastie tricuspide;
- La contrainte du coût dans notre pays ainsi que dans les pays en voie de développement, conduit la plupart des opérateurs à préférer la technique de deVega, cette étude vient de comparer deux techniques fréquemment pratiquées dans nos pays la plastie avec anneau de carpentier et celle de deVega et de démontrer que le cout ne doit pas influencer la technique même dans les pays en voie de développement;
- Cette étude a permis également de déterminer les facteurs prédictifs de récidive de la fuite dans notre population ce qui permet de guider l'opérateur dorénavant dans le choix de la technique, c'est à dire ceux qui ont présentent des facteurs de mauvais pronostic à long terme, devraient bénéficier d'emblée de la technique la plus performante quel que soit le coût.

## Conflits d’intérêts

Les auteurs ne déclarent aucun conflit d'intérêts.
